# interRAI Subjective Quality of Life Scale for Mental Health and Addiction Settings: A Self-Reported Measure Developed From a Multi-National Study

**DOI:** 10.3389/fpsyt.2021.705415

**Published:** 2021-07-09

**Authors:** Hao Luo, Alice Hirdes, Jyrki Heikkilä, Kathleen De Cuyper, Chantal Van Audenhove, Margaret Saari, John P. Hirdes

**Affiliations:** ^1^Department of Social Work and Social Administration, The University of Hong Kong, Hong Kong, China; ^2^Sau Po Centre on Ageing, The University of Hong Kong, Hong Kong, China; ^3^Hong Kong Jockey Club Centre for Suicide Research and Prevention, The University of Hong Kong, Hong Kong, China; ^4^Graduate Program in Health Promotion, Human Development and Society, Lutheran University of Brazil, Canoas, Brazil; ^5^Division of Psychiatry, Turku University Hospital, Turku, Finland; ^6^LUCAS Center for Care Research and Consultancy, Academic Center for General Practice, Department of Public Health and Primary Care, KU Leuven University, Leuven, Belgium; ^7^SE Research Centre, SE Health, Markham, ON, Canada; ^8^Lawrence S. Bloomberg Faculty of Nursing, University of Toronto, Toronto, ON, Canada; ^9^School of Public Health Sciences, University of Waterloo, Waterloo, ON, Canada

**Keywords:** interRAI, quality of life, mental health, staff relationship, psychometric properties, addictions

## Abstract

**Background:** Measuring Quality of Life (QoL) in mental health using self-reported items is important for evaluating the quality of service and understanding the person's experience of the care received.

**Objective:** The aim of this research was to develop and validate a self-reported QoL instrument for inpatient and community mental health settings.

**Methods:** Data were collected from diverse research sites in Canada, Belgium, Russia, Finland, Brazil, and Hong Kong, using the 37-item interRAI Quality of Life Survey for Mental Health and Addictions. The survey was administrated to 2,218 participants from inpatient and community mental health settings, assisted living, and the general community. We randomly divided the sample into a training and a test sample (70 and 30%, respectively). We conducted principal component analysis (PCA) and exploratory factor analysis (EFA) using the training sample to identify potential factor structure. Confirmatory factor analysis (CFA) models were then fitted to finalize and externally validate the measurement model using training and test data, respectively.

**Results:** PCA, EFA, and CFA of the training sample collectively suggested a 23-item scale measuring four latent constructs: well-being and hope (8 items), relationship (7 items), support (5 items), and activity (3 items). This model was supported by the CFA of the test sample. The goodness-of-fit statistics root mean square error, comparative fit index and Tucker-Lewis index were 0.03, 1.00, and 0.99, respectively. Estimated Cronbach's alpha based on the test data was 0.92. Raw Cronbach's alpha values for the subscales were 0.86 for well-being and hope, 0.86 for relationship, 0.69 for support, and 0.72 for activity.

**Conclusions:** The interRAI SQoL-MHA scale is a valid instrument to measure QoL in mental health settings. The instrument will support the evaluation of the quality of care and can also be used for future research to produce SQoL-MHA values on a quality adjusted-life-year scale, facilitating the evaluation of various mental health interventions.

## Introduction

The past two decades have witnessed a major shift in mental health service policy from an emphasis on symptom reduction to a holistic consideration of recovery, social functioning, and quality of life (QoL) (1). In its Mental Health Action Plan 2013–2020, the World Health Organization has listed QoL as one of the crucial information indicators for the mental health system. The need to disaggregate mental health outcomes by subpopulations to reflect the diverse needs of individuals with different socio-economic and clinical characteristics has been highlighted (2). Another growing consensus is the importance of incorporating perspectives of mental health service users in evaluating clinical trials, services, and policies. Consequently, the interest in patient-reported measures of QoL has gained prominence in mental health practice (3–5).

Several approaches exist for measuring QoL, including objective approaches, subjective approaches, and health-related QoL (HRQoL) (6). One of the earliest approaches, the objective approach, focuses on life circumstances such as employment, income, and housing status (7). Objective measures of health and functioning status are also included (8). However, the objective approach can be limited since patients with similar clinical characteristics and life circumstances can exhibit dramatically different behavioral and emotional responses (6). Subjective quality of life focuses more on people's satisfaction and happiness (9). Early studies of people with mental health difficulties identified a common range of domains, including subjective appraisals of work, leisure, social relationships, finances, health, environment, and opportunities for self-fulfillment (10). Although the objective and subjective perspectives do not always coincide, they both represent important aspects of quality of life that cannot be ignored. HRQoL in essence refers to an individual's perceived physical and mental health over time. Numerous generic, i.e., applicable to the whole population, or disease-specific HRQoLs have been developed. Owing to the wide adoption of cost-effective analysis to inform resource allocation in health care, generic preference-based measures represented by EuroQoL-5D (EQ-5D) and SF-6D have become the most commonly used instruments worldwide (11). Both EQ-5D and SF-6D include a health state descriptive system and a utility scale used to calculate quality-adjusted life years (QALYs). In particular, EQ-5D has been endorsed by the National Institute for Health and Care Excellence (NICE) in England (12).

The question of whether generic measures EQ-5D and SF-6D are “fit for purpose in mental health” was raised more than a decade ago (13). Due to the substantial increase in the use of cost utility analysis, various studies have examined the psychometric validity of EQ-5D and SF-6D in respect to different mental health conditions. Although both measures have demonstrated acceptable levels of construct validity and responsiveness in common mental health problems (such as depression), mixed diagnoses, and personality disorder, a low level of construct validity and responsiveness is evident in anxiety disorders, schizophrenia, and psychosis (14–18). A large-scale study which examined the psychometric validity of EQ-5D and SF-6D displayed a low level of sensitivity and relationship with a wide range of condition-specific indicators (11). This evidence collectively highlights the need to develop QoL measures that are specific to mental health settings, especially for the purpose of making clinical decisions, assess health changes over time, or evaluate the quality of mental health services.

QoL is affected by the complex interactions of factors across the life course. Although QoL is not a characteristic of interventions, treatment, or services a person receives, previous studies have shown a significant relationship between changes in QoL and the quality of care (19). Relationships with mental health professionals are not simply based on technical or procedural transactions, so it is reasonable to expect that those relationships are a meaningful dimension of the person's subjective experience of daily life. We hence hypothesize that relationships with staff and access to service are important correlates of mental health service users' quality of life.

The interRAI Self-Reported Quality of Life Survey for Mental Health and Addictions was developed by interRAI, an international non-profit network of more than 100 clinicians and researchers from over 35 countries (20). The network has developed standardized assessment tools for use in various areas of heath, including mental health (20). These tools can help to provide population-based data as an input to policy decision-making, as well as provide better care plans for individuals and to make best use of available funding. The interRAI Mental Health (MH) and Community Mental Health (CMH) assessment systems were developed to provide a comprehensive assessment of the strengths, preferences, and needs of all adults in inpatient and community mental health settings (20). In addition to large-scale implementation of interRAI mental health instruments in Canada, Belgium, and Switzerland, pilot studies are being undertaken in Finland, Russia, Brazil, and Hong Kong. These assessor-rated instruments allow a service provider to assess key domains of functioning, mental and physical health, social support, and service use. The objective dimension of QoL is also included (21, 22). To supplement existing instruments that are designed for use by mental health professionals such as nurses, social workers, case managers, psychiatrists, and psychologists, the interRAI Self-Reported Quality of Life Survey for Mental Health and Addictions was developed to incorporate the individual's perspective to reflect changes experienced by mental health service users.

The development of the interRAI Self-Reported Quality of Life Survey for Mental Health and Addictions was guided by the literature on recovery from mental illness, where recovery can be defined as “the establishment of a fulfilling, meaning life and a positive sense of identity founded on hopefulness and self-determination” (23, 24). Key processes of recovery include: (1) finding and maintaining hope, (2) re-establishing a positive identity, (3) finding meaningful in life, (4) taking responsibility for one's life, and (5) connectedness (25). As far as we are aware, the development of only one scale, the Recovering Quality of Life scale (ReQoL), was guided by the recovery theme (12). Both REQoL-10 and ReQoL-20 have been shown to be appropriate for measuring service-user recovery-focused QoL outcomes based on the data from the United Kingdom. However, these scales do not consider the psychosocial dimension (e.g., relationship with friends and families).

The interRAI family of assessment instruments considers QoL as a multidimensional concept that includes both the objective and subjective domains and addresses a much broader range of a person's experience than HRQoL. Specifically, the self-reported QoL survey focuses on the subjective domain and aims to allow mental health service users to express their own views about their lives. In developing the survey, feedback from clinical staff and mental health service stakeholders in several countries, as well as inputs from the interRAI Network of Mental Health, which comprises an international and multidisciplinary team of academics, clinicians, and psychometricians, were also sought. The final set of items covers 10 domains: (1) personal outlook, (2) autonomy and self-determination, (3) meaningful activities, (4) friends and family, (5) community, (6) staff relationship, (7) privacy, (8) empowerment and support, (9) discrimination and life circumstances, and (10) access to service. The full list of items under each domain is provided with an accompanying training manual (26). A pilot study of 83 inpatients from a mental health center in Ontario, Canada provided a preliminary examination of the reliability of the interRAI Self-Reported Quality of Life Survey for Mental Health and Addictions. The resulting Cronbach's alpha values for the 10 domains were moderate to high (27). The pilot study concluded that the analyses were only provisional and called for further research including more respondents, in more diverse settings, and in different countries to further test the reliability and validity of the instrument.

To help staff in mental health settings create care plans that are meaningful to the individual, the primary objective of this study was to develop a self-reported QoL for mental health and addictions (SQoL-MHA) scale, using pooled data collected from a multi-regional study conducted in six countries or territories. In addition, a Staff Relationship Scale was created to help service providers identify areas for improvement. The construction of both scales was based on items from the interRAI Self-Reported Quality of Life Survey for Mental Health and Addictions (27), a complementary tool to the assessor-rated assessment instruments developed by the interRAI research group (20).

## Materials and Methods

### Data Source

In this multi-regional study, surveys were administrated by trained interviewers from Canada, Belgium, Finland, Russia, Brazil, and Hong Kong over different periods between 2010 and 2020 (see Supplementary Material for details). The initial sample comprised 2,218 respondents, 701 (31.61%) from community mental health settings, 725 (32.69%) from inpatient settings, 148 (6.67%) from transitional care, and 644 (29.94%) recruited from the general community. The sample sizes from each study site by setting are shown in Table 1. Staff measures were administrated to persons who were using mental health services. Data from this subsample of 1,574 were used to develop the Staff Relationship Scale.

**Table 1:** Relative frequency table of data sources by setting (*N* = 2,218).

	**Community mental health**	**Inpatient**	**General community**	**Transitional care**
Canada – community			644	
Canada – inpatient 1		87		
Canada – inpatient 2		83		
Canada - transitional care				148
Belgium	234	181		
Russia		200		
Finland		174		
Brazil	412			
Hong Kong	55			
Total	701	725	644	148

Ethical approval was obtained from the Office of Research Ethics (ORE) at the University of Waterloo (ORE#13848, ORE#20863) for the Canadian, Finnish, Russian, and Hong Kong samples; Southlake Regional Health Centre Ethics Board (SRHC REB) (#0006-1819) for the Canadian transitional care sample; Ethical Committee Research from Centro Universitário São Lucas Ji-Paraná (CAAE 29517319.9.0000.5297) and Ethical Committee Research from Universidade Luterana do Brasil (CAAE 60213316.9.0000.5349) for the Brazilian sample; and Ethical Committee Research of KU Leuven – University of Leuven (Belgium) (S61488) for the Belgian sample.

### Measures

The interRAI Self-Reported Quality of Life Survey for Mental Health and Addictions comprises 37 items measuring the person's quality of life and experience with mental health services. The survey has two overarching aims to: (1) learn what life is like for the person; and (2) examine how well a program is providing services to the person. For each item, the respondent is asked to answer on a five-point Likert scale: 0 (Never), 1 (Rarely), 2 (Sometimes), 3 (Most of the time), and 4 (Always). This scale was then collapsed into a three-point scale of 0–1 (Never or rarely), 2 (sometimes), and 3–4 (most of time or always) due to low responses in the “Never” and “Rarely” categories. Twenty-seven items were administrated to all participants and were used as candidate items for developing the SQoL-MHA scale. The remaining 10 items were administrated to mental health services users and used as candidate items for developing the Staff Relationship Scale.

### Analytical Procedure

We adopted the validation set approach for developing the SQoL-MHA scale. The total sample was randomly divided into a training sample consisting of 70% of the total observations and a test sample of 30%. Our preliminary analysis showed that percentages of missing values ranged from 2 to 8% in the training sample. Using the training data, we first examined the traditional item psychometric properties, represented by item-total correlations and the non-missing response frequency for each item. Items with item-total correlations <0.4 or missing values more than 5% were removed from the subsequent analyses. Our preliminary analysis showed that most items had item-total correlations >0.5. We then chose a more stringent value of 0.4 (slightly higher than the commonly adopted value of 0.3) as the cut-off value to ensure the final scale measuring a general construct QoL (28). A high missing rate in an item may indicate problems such as the items were being poorly worded, exceeding the reading skills of the respondents, or being too specific to a living situation or a diagnosis. In practice, a missing value rate of 5% or above, as a rule of thumb, often requires imputation (29). At the scale development stage, we again chose a strict cut-off value of 5% to rule out potentially problematic items. For the remaining items, the listwise deletion technique was used for handling missing values.

**Figure 1:**
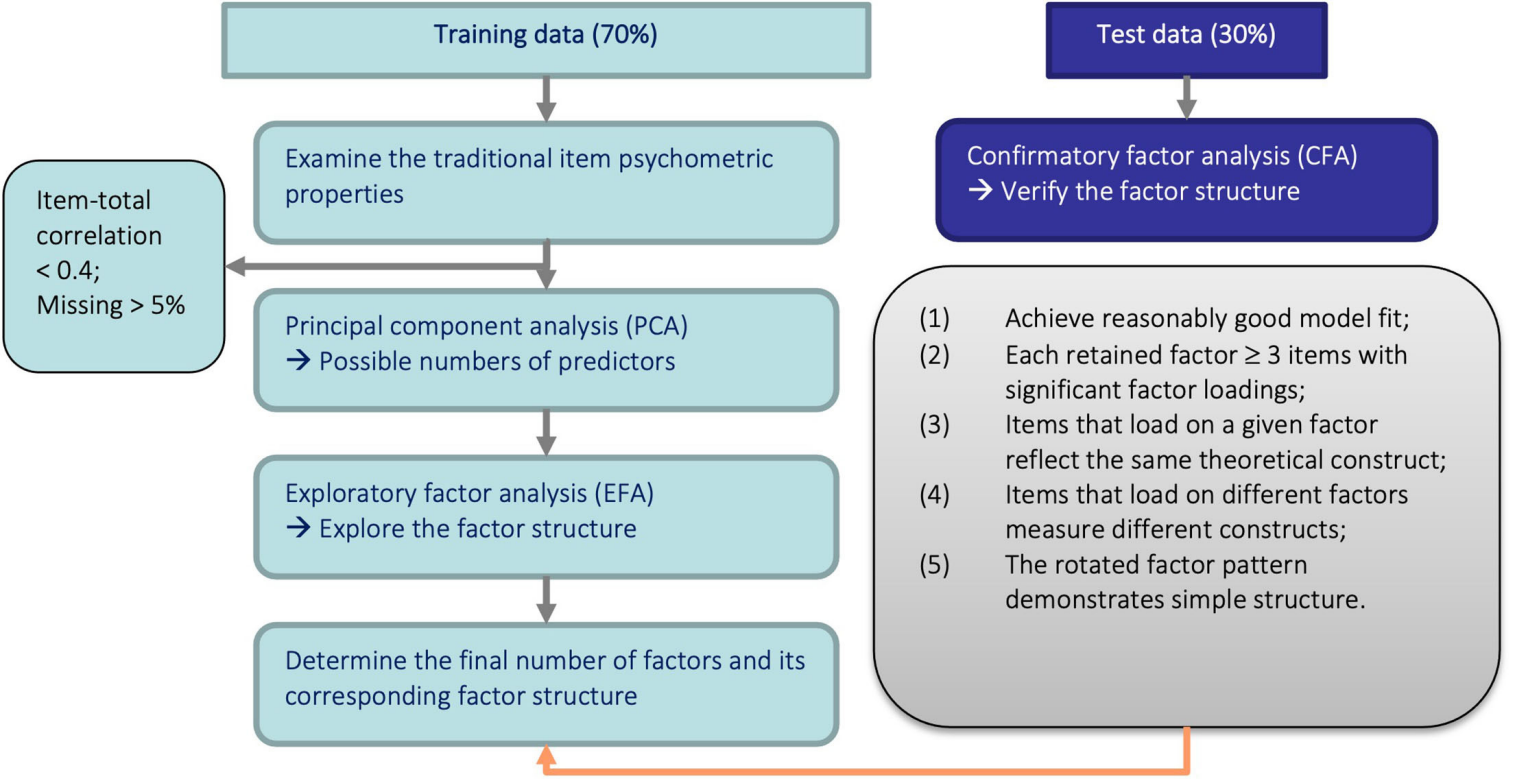
Illustration of the analytical procedure.

We performed principal component analysis (PCA) to identify the possible number of factors indicated by the number of components with eigenvalues greater than one. We then fitted several exploratory factor analysis (EFA) models to investigate the potential factor structures. The number of components identified in PCA was compared to the number of factors indicated by the best model in EFA for consistency check. Note that although PCA is a descriptive model of data that attempts to account for the entire variance of the correlation matrix rather than just the common variance as in EFA, the number of principle components with eigenvalues greater than one should not deviate substantially from the number of factors indicated by the best fitting EFA model (30). For the EFA, a standard deviation of the residuals (RMSR) <0.05 and a goodness-of-fit measure equal or higher than 0.9 were considered a good fit (31). Determination of the final number of factors and their corresponding factor structure was based on five criteria: (1) EFA model achieves reasonably good model fit; (2) each retained factor is measured by at least three items; (3) items that load on a given factor reflect the same theoretical construct; (4) items that load on different factors measure different constructs; and (5) the rotated factor pattern demonstrates simple structure, i.e., each item should measure a unique domain. Items that did not have sufficiently large loadings on any factors were removed. The resultant factor structure was evaluated in both the training and test samples using confirmatory factor analysis (CFA). This analytical procedure for developing the SQoL-MHA scale is illustrated in Figure 1.

The same steps were applied to the development of the Staff Relationship Scale except that we did not use the validation set approach, as the sample size of the service user population was smaller than the general population, and splitting the sample may have comprised the power of the subsequent analysis. The scale was developed and validated using the same sample with complete cases.

### Estimation Methods and Fit Statistics

Since responses for all items are ordinal variables, polychoric correlation (instead of Pearson's correlation) coefficients were computed for all data points for latent variable model fittings. For EFA, the principal axis factoring with oblique oblimin rotation was used together with polychoric correlation (32). For CFA, polychoric correlation was used in combination with the diagonally weighted least squares (DWLS) estimator. Previous simulation studies have demonstrated the superior performance of this approach (33). Root mean square error (RMSEA), comparative fit index (CFI), and Tucker-Lewis index (TLI) were used to evaluate the model goodness-of-fit. The cut-off criteria of these goodness-of-fit statistics are heavily contingent. In this study, RMSEA <0.08, CFI > 0.95, and TLI > 0.95 were considered as a reasonable model-data fit (34–36). However, it is important to note that these cut-off values were concluded based on continuous data that were analyzed using normal-theory maximum likelihood (ML) and should be applied with caution when being generalized to ordinal data that were analyzed using polylchoric correlation and estimators other than ML (37). All data analysis was conducted using the statistical software R (38). Specifically, the *psych* package was used for PCA and EFA and the *lavvan* package was used for CFA (30, 39).

## Results

Of the 1,937 respondents with a valid record of gender, 1,041 (53.74%) were female and 896 (46.26%) were male. The majority were in the under 45 and 45–64 years age groups, accounting for 46.49 and 33.02%, respectively, of the 1,781 respondents with a valid record of age. The percentages of respondents aged 65–74 and 75 years or older were 13.81 and 6.79%, respectively.

### Development of the interRAI SQoL-MHA Scale

Traditional item psychometric properties for the training sample, including the response proportions and item-total correlation, are summarized in Table 2. Raw and standardized Cronbach's alphas generated from the training sample were both 0.90. Item-total correlations ranged from 0.15 (“satisfied with services”) to 0.68 (“on the whole, life is good”). One item (opportunities for work or school) was removed due to excess missing values (>5%) and three were removed due to low item-total correlation (<0.4).

**Table 2:** Abbreviated item content and traditional item psychometric properties: training sample (*N* = 1,552).

**Item**	**Missing%**	**Response proportion%**			**Item-total correlation**
		**0**	**1**	**2**	
Safe w/family and friends	3	9	14	78	0.61
Safe and comfortable in home	3	9	11	80	0.53
If I need help right away, I can get it	2	8	17	75	0.48
In a crisis, know where to get help	3	9	15	77	0.51
Hopeful about future	3	9	24	67	0.62
Life getting better	4	11	30	59	0.63
Feel good about myself	2	12	25	63	0.65
On the whole, life is good	3	12	21	67	0.68
Have good place to live	3	10	10	80	0.53
Manage stresses in life	3	15	29	56	0.58
Know how to make life better	4	12	26	63	0.60
Make choices about things that matter	4	9	19	72	0.57
Concerned about how others treat me	4	32	31	38	0.36
Worried about making ends meet^a^^,^^b^	4	40	25	35	0.38
Can get health services^a^^,^^b^	2	5	14	81	0.44
Satisfied with services^b^	5	4	14	81	0.15
Participated in meaningful activities	3	15	26	59	0.51
Opportunities for work or school^c^	8	31	14	54	0.45
Motivated in day to day activities	3	12	24	63	0.61
Participate in community activities	2	39	31	30	0.46
Important role in people's lives	4	11	21	68	0.61
Friends and family believe in me	3	9	17	74	0.61
Relationships are good	2	9	15	77	0.58
Help family and friends	3	7	14	79	0.49
Feel part of neighborhood	3	19	24	57	0.52
Get support for decisions	3	8	17	75	0.55
Have people I can count on	2	6	13	81	0.59

a *Reversed item;*

b *Removed due to low item-total correlation;*

c *Removed due to excess missing values*.

We then performed PCA on the remaining 23 items. A total of 1,289 participants in the training sample that did not have missing values in any of the 23 items were included in the subsequent analysis. The first five eigenvalues from PCA ranged from 10.67 to 1.03, suggesting that four to five factors were likely to be sufficient. The eigenvalue ratio of the first (10.67) to second (1.87) eigenvalues was 5.71 (>3), suggesting that a unidimensional interpretation of the scale, in this case meaning that the scale measures a dominant latent construct of quality of life, is appropriate.

EFA models of up to five factors were fitted to investigate the potential factor structure. The four- and five-factor models provided the best fit with the data, with RMSRs of 0.04 and 0.03, and goodness of fit of off diagonal values of 0.99 and 1.00, respectively. However, only two items loaded to the fifth factor in the five-factor model. The four-factor EFA model was then chosen as being superior overall based on our analytical protocol (Figure 1). Standardized loadings (pattern matrix) of the model based on 23 items are shown in Table 3. Factor 1 was predominantly measured by eight items: (1) hopeful about future, (2) life getting better, (3) feel good about myself, (4) on the whole, life is good, (5) manage stresses in life, (6) know how to make life better, (7) make choices about things that matter, and (8) motivated in day-to-day activities, which correspond to a more general construct of well-being and hope. Factor 2 was measured by seven items, including (1) safe with family and friends, (2) safe and comfortable in home, (3) have good place to live, (4) important role in people's lives, (5) friends and family believe in me, (6) relationships are good, and (7) help family and friends, which concurred with the theoretical construct relationship. Factor 3 was measured by five items: (1) if I need help, I can get it, (2) in a crisis, know where to get help, (3) can get health services, (4) get support for decisions, and (5) have people that I can count on, that are in line with the construct support. Factor 4 was measured by 3 items: (1) participated in meaning activities, (2) participate in community activities, and (3) feel part of a neighborhood, which correspond to the activity domain. The factor with the higher loading was kept for the subsequent CFA.

**Table 3:** Estimation results of the four-factor exploratory factor analysis model: training sample (*N* = 1,289).

	**PA1**	**PA2**	**PA3**	**PA4**	**h2^1^**
Safe w/family and friends		0.89			0.81
Safe and comfortable in home		0.63			0.51
If I need help right away, I can get it			0.86		0.70
In a crisis, know where to get help			0.71		0.57
Hopeful about future	0.82				0.68
Life getting better	0.75				0.62
Feel good about myself	0.82				0.69
On the whole, life is good	0.59	0.38			0.70
Have good place to live		0.63			0.52
Manage stresses in life	0.61				0.48
Know how to make life better	0.78				0.60
Make choices about things that matter	0.55	0.25			0.48
Can get health services			0.79		0.58
Participated in meaningful activities	0.37			0.42	0.45
Motivated in day to day activities	0.63			0.27	0.60
Participate in community activities				0.74	0.63
Important role in people's lives		0.64			0.61
Friends and family believe in me		0.78			0.72
Relationships are good		0.89			0.79
Help family and friends		0.37			0.41
Feel part of a neighborhood				0.50	0.47
Get support for decisions	0.24		0.48		0.53
Have people that I can count on		0.37	0.43		0.64

1 *Communalities*.

*Factors loadings <0.2 were are not shown here*.

Using the same training sample, we investigated the factor model by conducting a separate CFA, allowing each item to load on only one factor to ensure a simple structure. The Chi-square test statistic was 1049.47 with degrees of freedom of 224. The RMSEA was 0.05 (90% CI: 0.05–0.06) and the SRMR was 0.07. CFI and TLI were 0.99 and 0.98, respectively. This set of goodness-of-fit statistics collectively suggested that the model fit the data well. The factor loadings are summarized in Table 4. Factor loadings are all fairly large, suggesting that they are all sufficiently good measures of the respective latent variables. Correlations among the four factors ranged from 0.29 to 0.50 (Table 5), which suggests that the four factors measured correlated, yet distinct constructs.

**Table 4:** Estimation results from Confirmatory Factor Analysis.

**Domain**		**Training sample**			**Testing sample**		
		**(*****N*** **= 1,289)**			**(*****N*** **= 550)**		
	**Item**	**Est**.	**SE**	**α**	**Est**.	**SE**	**α**
Well-being	Hopeful about future	1		0.86	1		0.86
& Hope	Life getting better	0.96	0.02		0.98	0.03	
	Feel good about myself	1.01	0.02		1.06	0.03	
	On whole, life is good	1.05	0.02		1.09	0.03	
	Manage stresses in life	0.86	0.02		0.90	0.03	
	Know how to make life better	0.91	0.02		0.93	0.03	
	Make choices about things that matter	0.87	0.02		0.92	0.03	
	Motivated in day-to-day activities	0.90	0.02		0.99	0.03	
Relationship	Safe w/family and friends	1		0.84	1		0.86
	Safe and comfortable in home	0.87	0.02		0.95	0.03	
	Have good place to live	0.87	0.02		0.90	0.03	
	Important role in people's lives	0.91	0.02		0.96	0.03	
	Friends and family believe in me	1.00	0.02		1.05	0.03	
	Relationships are good	1.00	0.02		1.08	0.03	
	Help family and friends	0.78	0.02		0.86	0.03	
Support	If I need help right away, I can get it	1		0.75	1		0.69
	In a crisis, know where to get help	1.02	0.03		0.99	0.06	
	Can get health services	0.91	0.03		0.92	0.05	
	Get support for decisions	1.15	0.03		1.38	0.06	
	Have people that I can count on	1.33	0.03		1.51	0.07	
Activities	Participated in meaningful activities	1		0.61	1		0.72
	Participate in community activities	0.89	0.03		0.854	0.03	
	Feel part of a neighborhood	1.02	0.03		0.979	0.03	

*All factor loadings have P-values < 0.001*.

**Table 5:** Correlation matrix of latent variables.

	**Training sample (*****N*** **= 1,289)**			**Testing sample (*****N*** **= 550)**		
	**Hope**	**Relationship**	**Support**	**Hope**	**Relationship**	**Support**
Relationship	0.50 (0.01)			0.52 (0.02)		
Support	0.39 (0.01)	0.40 (0.01)		0.30 (0.01)	0.38 (0.02)	
Activities	0.41 (0.01)	0.39 (0.01)	0.29 (0.01)	0.47 (0.02)	0.46 (0.02)	0.30 (0.02)

*All factor loadings have P-values <0.001*.

To examine external validity of the factor structure of the QoL scale, the model was fit to a test sample of 550 respondents (see also Table 4 for the estimation results). The Chi-square statistic was 366.93 (df = 224). The RMSEA and SRMR were 0.03 (90% CI: 0.03–0.04) and 0.06, respectively. CFI was 1.00 and TFI were 0.99. Raw and standardized Cronbach's alphas generated from the test sample were both 0.92. Raw Cronbach's alpha values for the subscales were 0.86 for well-being and hope, 0.86 for relationship, 0.69 for support, and 0.72 for activity. We also conducted a sensitivity analysis to examine the impact of removing items with potential cross loading problem on the CFA model fit and sub-domain reliabilities. Only one item, “have people that I can count on,” had relatively high loadings of 0.37 and 0.43 on two factors based on the EFA. Removing this item had no effect on all goodness of fit statistics of interest except that SRMR decreased by 0.001 in the training sample. However, the reliability of the support sub-domain decreased from 0.75 to 0.71 in the training sample, and from 0.69 to 0.62 in the testing sample. This item was therefore kept in the final SQoL-MHA scale.

We further examined the reliabilities by sub-domains for the total sample (combining training and testing data). Raw and standardized Cronbach alphas generated from the total sample were both 0.91. Sub-scale Cronbach alpha values ranged from 0.64 for the activity domain to 0.86 for the well-being and hope domain.

### Development of the Staff Relationship Scale

Table 6 shows the response patterns and item-total correlations of the 10 staff items. Raw and standardized Cronbach's alpha values were 0.78 and 0.81, respectively. The percentage of missing values ranged from 4 to 10%. The “feel valued and respected” item was removed due to its lower item-total correlation and the “personal information kept private” item was removed due to excessive missing values (10%). Raw and standardized Cronbach's alpha values increased to 0.81 and 0.82, respectively. We did not strictly follow the missing value <5% and item-total correlation >0.4 rule here since only two items had missing values <5%.

**Table 6:** Abbreviated item content and traditional item psychometric properties for subsample of service users.

**Item**	**Missing%**	**Response proportion%**			**Item-total correlation**
		**0**	**1**	**2**	
Private conversation	7	7	12	81	0.67
Personal information kept private	10	6	6	89	0.59
Safe around those who provide care	4	3	11	86	0.58
Treated with respect	4	3	8	89	0.59
Feel valued and respected	5	21	28	51	0.48
Privacy respected by staff	7	3	7	89	0.59
Staff help me take responsibility	6	5	16	78	0.63
Can speak my mind around staff	5	7	15	78	0.70
Staff listen to what I say	5	4	15	82	0.67
Staff support my recovery	6	2	10	88	0.65

The PCA showed that only the first eigenvalue of 5.05 was greater than one, suggesting that one factor might be sufficient. The two-factor EFA model failed to converge, which also pointed to a single factor model. We then constructed an 8-item single factor CFA model using data from 1,341 complete cases. The model had a Chi-square statistic of 165.04 (df = 20), RMSEA of 0.07, CFI of 0.97, and TLI of 0.95. The factor loadings for the CFA are shown in Table 7.

**Table 7:** Confirmatory Factor Analysis estimation results for the Staff measure (*N* = 1,341).

	**Estimate**	**Standard errors**
Private conversation	1	
Safe around those who provide care	1.05	0.05
Treated with respect	1.02	0.06
Privacy respected by staff	1.07	0.06
Staff help me take responsibility	1.09	0.06
Can speak my mind around staff	1.15	0.06
Staff listen to what I say	1.22	0.05
Staff support my recovery	1.24	0.05

*All factor loadings have P-values <0.001*.

## Discussion

In this study, we report the process of developing and validating the SQoL-MHA scale, a new measure for assessing the subjective QoL for mental health service users. The SQoL-MHA is a concise 23-item scale measuring four domains of QoL: well-being and hope, relationship, support, and activity, that are measured by eight, seven, five, and three items, respectively. The CFA yielded a good fit of the test data and confirmed the four-factor model suggested by the EFA. The reliabilities of the sub-scales were moderate to high. The total score of the SQoL-MHA scale ranges between 0 and 46. Sub-scale score ranges were 0–16 for well-being and hope, 0–14 for relationship, 0–10 for support, and 0–6 for activity. The four domains included in the SQoL-MHA scale share similar aspects in key processes of recovery such as hope, self-determination, connectedness, and meaningful activities. The sub-scales can be used to assess specific domains of QoL of interest and to identify areas of improvement that should be targeted on. To allow mental health service providers identify specific areas for improvement and adapt their care environments to enhance users' QoL, an eight item Staff Relationships Scale was developed.

In contrast to scales that have been developed from a single country or region with a relatively homogeneous cultural background, the SQoL-MHA scale was developed through a collective effort of partners in the interRAI family from six countries across four continents. The study involved diverse service environments including inpatient psychiatry, community mental health, and general community settings. To more effectively identify and respond to mental illness and related dimensions of health and well-being throughout the life course, the interRAI suite of mental health instruments has been designed as an integrated assessment and screening system providing a holistic view of an individual's strengths, preferences, and needs. A specific goal is to develop a common language for describing needs, monitoring service use, and tracking outcomes over time, across the health care continuum. It also advocates considering more than psychiatric symptoms alone by taking a broader perspective to address issues like growth, development, and aging; social relationships; economic resources; housing; stigma; and recovery. In the assessor-rated interRAI instruments (e.g., interRAI-MH and interRAI-CMH), objective dimensions of QoL can be measured by items from several relevant domains, including functional status, physical health conditions, social relations, employment, education, finance, and housing. Alongside the increasing implementation of interRAI mental health instruments across the world, the new SQoL-MHA and Staff Relationship Scale can be readily implemented in places where standard assessments have been routinely performed. These patient-reported measures of outcomes and experience of care provide an important subjective complement to existing assessor-rated instruments.

Compared with generic health-related quality of life measures like EQ-5D and SF-6D, the SQoL-MHA scale offers significant advantages as a measure developed specifically for mental health service users. It should also function better than condition-specific measures such as the Hospital Anxiety and Depression Scale (HADS) and the Patient Health Questionnaire-9 (PHQ-9) since it considers a broader range of users with mental health conditions beyond those with depression and anxiety.

This study has some several limitations. First, our respondents were not randomly selected and were unevenly distributed across regions and settings. Any generalization of the findings needs to be made cautiously. Limited by relatively small sample sizes in Finland, Russia, and Hong Kong, measurement invariance analysis was not conducted. It is not clear whether certain items would function differently by culture or characteristics other than the latent construct of interest. Future work should investigate group-wise measurement invariance by subpopulations and longitudinal measurement invariance. The interRAI Self-Reported Quality of Life Survey for Mental Health and Addictions was administrated without additional data on other scales. Therefore, we did not examine the known-group validity due to the lack of diagnostic information in the pilot sites. Neither did we compare QoL across regions as the sample sizes varied significantly between different sites. Only reliability, face-validity, and construct-validity can be established based on the current data. However, a gold standard does not exist for measuring QoL in mental health and addictions. Although adding other scales in the future can help to better understand the difference between generic measures and SQoL-MHA, the absence of a gold standard measure makes it difficult to establish criterion validity. We focused only on examining the psychometric properties of the new measures. Future research is needed to administer generic and condition-specific measures in addition to the interRAI SQoL-MHA scale to better understand their performance with different mental health populations.

## Conclusion

The 23-item interRAI SQoL-MHA scale is a valid instrument to measure QoL in mental health settings. When used with the Staff Relationship Scale, it will support the evaluation of care quality. Combined with existing information collected through interRAI MH and interRAI CMH, a holistic view (including both the objective and subjective perspectives) of a person's QoL can be assessed. In addition, the tool can be used to calculate quality-adjusted-life years, which will facilitate the evaluation of various health intervention, treatments, and policies in mental health settings. Future research is planned to establish the weights metric needed for calculating QALYs.

## Data Availability Statement

The datasets presented in this article are not readily available because the ethical approvals do not allow sharing of raw data of this project. Requests to access the datasets should be directed to John P. Hirdes, hirdes@uwaterloo.ca.

## Ethics Statement

The studies involving human participants were reviewed and approved by The Office of Research Ethics (ORE) at the University of Waterloo. Written informed consent for participation was not required for this study in accordance with the national legislation and the institutional requirements.

## Author Contributions

HL formulated the research questions, analyzed the data, and took the lead in writing the article. AH, JH, KD, CV, and MS collectively designed the study, lead the data collection in each study site, critically reviewed the analysis, and contributed to the writing of the article. JPH formulated the research questions, designed the study, coordinated the data collection, reviewed the analysis, and wrote the article. All authors contributed to the article and approved the submitted version.

## Conflict of Interest

The authors declare that the research was conducted in the absence of any commercial or financial relationships that could be construed as a potential conflict of interest.
