# Supplementary material for: interRAI Subjective Quality of Life Scale for Mental Health and Addiction Settings: A Self-Reported Measure Developed From a Multi-National Study
Source: Front Psychiatry. 2021 Jul 9;12:705415. doi: 10.3389/fpsyt.2021.705415 (PMC8298814; doi:10.3389/fpsyt.2021.705415)
Supplement: Supplementary file 1 [file Table_1.DOCX]

# Supplementary material. Quality of Life samples: site description, ethics approval and data collection period

- Canadian samples:
  - General community sample
    - Site description: telephone survey administered to randomly selected adults in Waterloo Region, Ontario, Canada. Only relevant interRAI QoL Mental Health and Addiction items were asked, nothing related to staff or service provision.
    - Ethics approval: UWaterloo Office of Research Ethics (ORE# 13848)
    - Data collection period: Summer 2011
  - Inpatient mental health sample 1
    - Site description: Inpatient Mental Health unit of a hospital in Ontario, Canada. Volunteer sample of inpatients. Mixed approach to data collection: both self-administered and interview-based.
    - Ethics approval: UWaterloo Office of Research Ethics (ORE# 13848)
    - Data collection period: December 2010 – March 2011
  - Inpatient mental health sample 2
    - Site description: individuals discharged from two Inpatient mental health units in a large community hospital, in Waterloo Region, Ontario, Canada. It concerns an Acute Inpatient Mental Health and Addictions unit and a Specialized Mental Health unit (longer lengths of stay).
    - Ethics approval: UWaterloo Office of Research Ethics (ORE #20863)
    - Data collection period: July – Dec 2015
  - Transitional care sample
    - Site description: short-term transitional care program which utilizes a reactivation and reintegration model designed to focus each client, their family, and their care team on the goal of going home with the appropriate supports in place. The target population for the transitional care program includes three key populations at risk for delayed discharge from acute care settings, clients living with mental health needs, clients living with Alzheimer’s disease or related dementias, and medically frail older adults.
    - Ethics approval: Southlake Regional Health Centre Ethics Board (SRHC REB) #0006-1819
    - Data collection period: Jun 1^st^, 2018 to March 13^th^, 2020
- Belgian sample
  - Site description: The data were collected in 49 mental health care facilities in Flanders, Belgium. These included psychiatric nursing homes, sheltered house living services, inpatient and outpatient mental health rehabilitation services, inpatient and outpatient addiction services and mobile mental health teams. Patients with a severe and enduring mental illness (addiction included) completed the Quality of Life survey, some with help from a caregiver.
  - Ethics approval: Ethical Committee Research of KU Leuven – University of Leuven (Belgium) S61488
  - Data collection period: September 2019 to June 2020
- Finnish sample
  - Site description: Inpatient mental health unit from Helsinki hospitals.
  - Ethics approval: UWaterloo Office of Research Ethics  (ORE# 13848)
  - Data collection period: 2012
- Russian sample
  - Site description: Inpatient unit from two facilities in the St. Petersburg area
  - Ethics approval: UWaterloo Office of Research Ethics  (ORE# 13848)
  - Data collection period: 2012
- Brazilian sample
  - Site description: data were collected in two Psychosocial Care Centers (CAPS) and in Primary Health Care with participants of therapeutic groups from Gravataí, Rio Grande do Sul State, Brazil.  Additional data were collected in a regional CAPS that offers treatment to people with mental illness from 15 municipalities in Rondônia State, northern Brazil. The Psychosocial Care Centers, in their different modalities and types (adult with mental illness, child and youth, alcohol and drug users), offer daily care to patients in the intensive, semi-intensive and non-intensive modalities, with an interdisciplinary team that carry out the therapeutic planning in continuous evolution. The CAPS is integrated into the health care network and obeys the principles of universal access, integral care and equity of the Brazilian Unified Health System (SUS).
  - Ethics approval: (1) Ethical Committee Research from  Centro Universitário São Lucas Ji-Paraná , CAAE 29517319.9.0000.5297; (2) Ethical Committee Research from Universidade Luterana do Brasil, CAAE 60213316.9.0000.5349
  - Data collection period: June 2017 to September 2020
- Hong Kong sample
  - Site description: A Hong Kong community psychiatric rehabilitation service provider.
  - Ethics approval: UWaterloo Office of Research Ethics (ORE# 13848)
  - Data collection period: 2013
